# Discharge planning services for safe transition after hip fracture: a systematic review and meta-analysis of discharge readiness, functional recovery, and complication reduction

**DOI:** 10.7717/peerj.21270

**Published:** 2026-06-11

**Authors:** Chengli Yan, Danlei Zheng, Yuyu Chen, Kai Fang, Yanzhen Jin, Xiaoqi Ye, Weiwei Chu, Lili Yang

**Affiliations:** 1 Department of Nursing, the Fourth Affiliated Hospital of School of Medicine, and International School of Medicine, International Institutes of Medicine, Yiwu, Zhejiang, China; 2 Department of Information, The Fourth Affiliated Hospital of School of Medicine, And International School of Medicine, International Institutes of Medicine, Yiwu, Zhejiang, China

**Keywords:** Nursing, Discharge planning services, Hip fracture, Meta-analysis

## Abstract

**Background:**

The postdischarge period presents a high-risk transition for those frequently face debilitating challenges , including diminished self-care capacity, limited lower-extremity function, and a heightened risk of complications. Discharge Planning Services (DPS) are essential for mitigating these risks because they facilitate a coordinated and safe transition from hospital to home.

**Aim:**

To systematically evaluate the impact of structured discharge planning services ondischarge readiness, functional recovery, and complication reductionfor patients after hip fracture.

**Methods:**

We conducted a systematic review and meta-analysis of randomized controlled trials (RCTs). A comprehensive literature search was performed from database inception until March 5, 2026 across PubMed, Embase, Web of Science, Cochrane Library, Ovid, EBSCO, and three Chinese databases (CNKI, Wanfang, VIP). We included RCTs evaluating DPS for patients with hip fractures. Data were pooled using a random-effects model, and heterogeneity was assessed with the *I*^2^ statistic.

**Results:**

Our systematic review included a total of 9 studies involving 861 patients with hip fractures. Meta-analysis revealed that DPS significantly improved patients’ self-care capacity (SMD = 0.59, 95% CI [0.43–0.75], *P* < 0.001) and multiple dimensions of functional status. Specifically, DPS led to notable gains in the Harris Hip Score (MD = 8.21, 95% CI [7.26–9.16], *P* < 0.001), the Fugl–Meyer Assessment Scale (MD = 4.61, 95% CI [3.87–5.35], *P* < 0.001), and the Berg Balance Scale (MD = 6.05, 95% CI [3.77–8.34], *P* < 0.001). Furthermore, DPS was associated with a significant reduction in complication rates (OR = 0.37, 95% CI [0.19–0.71], *P* = 0.002). An increase in Readiness for Hospital Discharge Scale scores was observed but did not reach statistical significance (SMD = 2.13, 95% CI [−0.60–3.66], *P* = 0.060).

**Conclusion:**

The findings from this meta-analysis suggest that discharge planning services can be effective at improving key transition-of-care outcomes, such as self-care capacity, and functional recovery, and reducing postdischarge complications in individuals recovering from hip fracture.

## Introduction

Hip fracture is a serious injury that results in high rates of disability, mortality, and healthcare burden worldwide, particularly among the elderly population. The incidence of complications is the highest among the elderly individuals (Li, Deng & Liu, 2021; Yusoff, Mulud & Mohammadnezhad, 2022). While surgical treatment typically yields successful results in stabilizing the fracture, the subsequent transition from hospital to home presents a significant challenge. This period is marked by increased vulnerability, with elevated risks of postdischarge complications, difficulties in functional recovery, and high hospital readmission rates (Beaupre et al., 2017; Chaharbakhshi, Schmitt & Brown, 2021). To address these challenges, this study utilizes discharge planning services (DPS), which is a patient-centric, coordinated approach designed to ensure continuity of care (Gonçalves-Bradley et al., 2022a). Discharge planning services, as defined by the American Hospital Association (AHA) in 1983 (Agency for Healthcare Research Quality, 2023; Earl, Katapodis & Schneiderman, 2020), involve a centralized, coordinated, and multispecialty integration process. This process ensures the continuity of healthcare for patients upon discharge through the collaboration of healthcare professionals and primary caregivers (American Hospital Association, 1983). Currently, discharge planning services are well-established and widely utilized in various countries, including the United States of America (Barkemeyer, 2015), the United Kingdom (National Health Service (NHS) England, 2021), Japan (Tomura et al., 2017), and China (Lin et al., 2013). In fact, patients with hip fractures have different requirements for discharge plans because of factors such as their advanced age, multiple complications, and challenges in functional recovery (Gonçalves-Bradley et al., 2022b; Chaharbakhshi, Schmitt & Brown, 2021). Research has demonstrated that discharge planning services can effectively enhance the discharge preparation of elderly patients with hip fractures. This, in turn, increases their quality of life, limb function, and patient satisfaction (Khayya, 2016; Bhandari & Swiontkowski, 2017; Lin et al., 2009; Dan et al., 2022; Beaupre et al., 2005). However, certain studies have indicated that the effects of discharge planning services on mortality rates, health outcomes, and costs remain uncertain (Shepperd et al., 2013). Therefore, this systematic review and meta-analysis aims to explore the effects of discharge planning services on improving health outcomes and reducing complications among patients with hip fractures. While evidence supports the effectiveness of the Fracture Liaison Service (FLS) in improving long-term bone health outcomes (Yan et al., 2023), its impact on immediate postdischarge transitions remains less explored, and the role of the DPS in addressing short-term transition challenges has not been thoroughly evaluated. This study aims to bridge this gap by providing evidence supporting structured discharge planning as an essential component of hip fracture care.

## Methods

### Study design and registration

This systematic review and meta-analysis was conducted in accordance with the Preferred Reporting Items for Systematic Reviews and Meta-Analyses (PRISMA) guidelines (Moher et al., 2009). The study protocol was registered on PROSPERO (Registration number: CRD420251024328).

### Search strategy

A comprehensive search for randomized controlled trials (RCTs) comparing discharge planning services (DPS) to usual care for patients with hip fractures was conducted. A comprehensive literature search was conducted from the inception of each included database up to 5 March 2026. The search comprised the following electronic databases: PubMed, Embase, the Cochrane Central Register of Controlled Trials (CENTRAL), Web of Science, Ovid MEDLINE, EBSCO, China National Knowledge Infrastructure (CNKI), Wanfang, and VIP. The search strategy included Medical Subject Headings (MeSH) terms and free-text keywords related to two key concepts, the details are presented in Table 1. We meticulously tailored the search syntax for each database, including PubMed. We also manually screened the reference lists of all included studies and relevant review articles to identify any additional eligible publications.

**Table 1:** Literature search strategy.

**Search concept**	**Search terms**
Hip fracture	“Hip Fractures”, “Femoral Neck Fractures”, “Intertrochanteric Fractures”, “pertrochanteric fracture”, “subtrochanteric fracture”
Discharge planning	“Patient Discharge”, “Discharge Planning”, “Discharge Preparation Service”, “transitional care”, “post-discharge”, “care transition*”

### Eligibility criteria

Studies were included if they enrolled adult patients (≥18 years) diagnosed with any type of hip fracture. The experimental intervention of interest was a structured discharge Planning Service , defined as a coordinated process aimed at preparing the patient for transition from hospital to home or another care setting. This was compared against usual care or standard postoperative management which lacked a structured DPS. The primary outcome was discharge readiness, which was assessed using tools such as the Readiness for Hospital Discharge Scale (RHDS). Secondary outcomes of interest included functional recovery (*e.g.*, measured by the Harris Hip Score or Barthel Index), lower extremity function (*e.g.*, evaluated *via* the Fugl–Meyer Assessment or Berg Balance Scale), the incidence of complications (such as venous thromboembolism, infection, or pressure ulcers), hospital readmission rates, length of hospital stay, patient satisfaction, and fall rates.

Study design: Only RCTs were included.

The exclusion criteria included other publication types (*e.g.*, qualitative studies, review protocols, editorials), those without available full texts, studies with unextractable data, or those reporting no relevant outcomes.

### Study selection and data extraction

Literature review and data extraction were conducted independently by two reviewers (YCL and ZDL). First, titles and abstracts were screened against the eligibility criteria. The full texts of potentially relevant articles were subsequently retrieved and thoroughly assessed. Any disagreements between the two reviewers were resolved through discussion or, if needed, consultation with a third senior reviewer (CYY). A predesigned data extraction form was used to gather specific details from each study , such as the first author, publication year, country, sample size, patient characteristics (*e.g.*, mean age, and sex), components of the DPS intervention, control group management, outcome measures, and key results. In cases where critical data were missing or unclear, we reached out to the corresponding authors *via* email for clarification.

### Risk of bias assessment

YCL and ZDL utilized the Cochrane Collaboration Tool 1.0 (RoB 1.0) (Green & Higgins, 2011) to assess the risk of bias in randomized controlled trials (RCTs). Any discrepancies in the assessment were resolved through consensus or by a third reviewer (CYY).

### Data synthesis and analysis

Statistical analyses were performed using Review Manager (RevMan, version 5.4; The Cochrane Collaboration). For continuous outcomes, the mean difference (MD) was calculated when studies used the same measurement scale, and the standardized mean difference (SMD) was used when different scales were employed, interpreting effect sizes as follows: small (SMD ≥ 0.2), medium (SMD ≥ 0.5), and large (SMD ≥ 0.8) (Green & Higgins, 2011). For dichotomous outcomes, odds ratios (ORs) was calculated. The effect estimates are presented with their 95% confidence intervals (CIs). We assessed statistical heterogeneity among studies using the I^2^ statistic. If the I^2^ value is greater than 50%, we use a random-effects model for meta-analysis; otherwise, we opt for a fixed-effects model. If there were significant discrepancies,we intended to investigate their origins through subgroup or sensitivity analyses. The possibility of publication bias was assessed using funnel plots and Egger’s test if sufficient studies (*n* > 10) were available for an outcome. The threshold for statistical significance was set at *p* < 0.050.

## Results

### Study selection and characteristics

**Figure 1:**
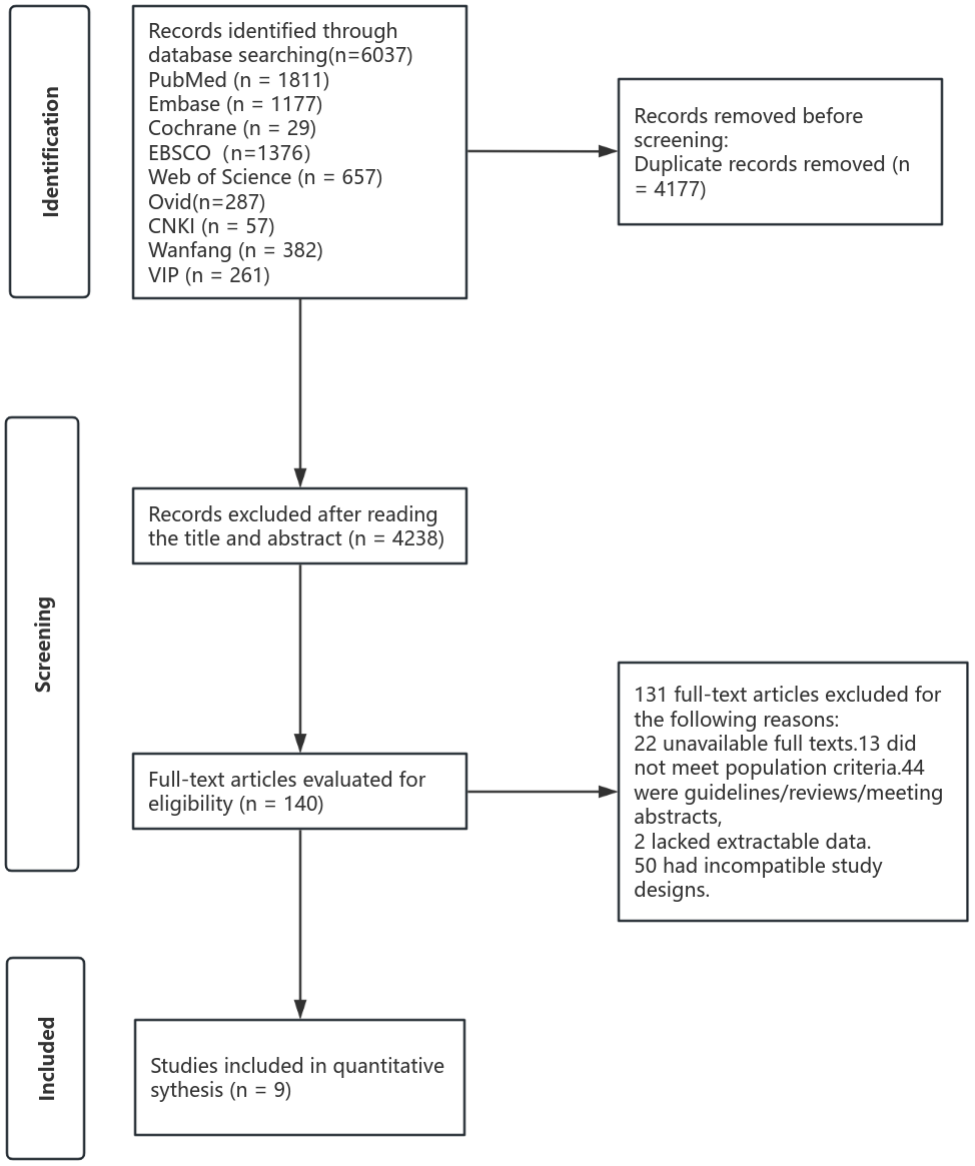
Flowchart of literature screening process.

The systematic search and selection process, detailed in the PRISMA flow diagram (Fig. 1), culminated in the inclusion of nine randomized controlled trials (Lin et al., 2009; Dan et al., 2022; Huang & Liang, 2005; Ying, Pan & Qiu, 2023; Lu et al., 2023; Ling, Sun & Qiu, 2022; Wang et al., 2023; Peng, Xu & Chen, 2023; Wang, 2021) for meta-analysis. These studies included a total of 861 patients with hip fractures. The key characteristics of each study including design, participant demographics, intervention specifics, and outcome measures, are summarized in Table 2.

**Table 2:** Characteristics of the included studies.

**Authors**	**Year**	**Age**		**Sample size**		**Interventions**		**Subjects**	**Appraisal time**	**Outcomes**
		**Control group**	**Trial group**	**Control group**	**Trial group**	**Control** **group**	**Trial** **group**			
Lin et al. (2009)	2009	78.75 ± 6.99	78.75 ± 6.99	24	26	1. Non-structured discharge instructions 2. No standardized process	1. Assessment of discharge needs 2. Personalized discharge care 3. Health education 4. Self-care 5. Identify referral sites to assist with referrals 6. Follow-up tracking	Hip fracture patients	Before intervention, after intervention, 2 weeks and 3 months after intervention	①②⑤⑧
Dan et al. (2022)	2022	69.71 ± 2.72	69.36 ±2.66	40	40	1. Perioperative care (life assistance, rehabilitation guidance, indicator management) 2. Discharge guidance (wound care, nutrition, exercise, medication) 3. Follow-up	1. Implementation discharge plan 2. Set up a discharge planning service team 3. Compile management manual 4. Assessment of discharge needs 5. Health education and rehabilitation guidance 6. Emotional management and safety management 7. Prevention of complications 8. Provide guidance to caregivers 9. Identify referral sites to assist with referrals 10. Establish a group of patients, Follow-up tracking	Elderly hip fracture	After intervention, 3 months after intervention	①③④⑥
Huang & Liang, (2005)	2005	78.1 ± 7.5	75.9±7.6	63	63	1. Nurses provide routine discharge planning 2. No brochures or written discharge summaries 3. No home visits or phone contact	1. Established navigation nurse 2. Team cooperation (Doctors, nurses, and family caregivers work together) 3. Assessment of discharge needs 4. Develop a personalized discharge plan 5. Diversified health education 6. Self-care and safety nursing 7. Follow-up tracking	Elders with hip fracture	After intervention, 2 weeks and 3 months after intervention	①②⑤⑦⑩
Ying, Pan & Qiu (2023)	2023	50.32 ± 1.33	50.29 ± 1.35	25	25	1.Health education (knowledge, postoperative precautions, lifestyle guidance) 2. Discharge (distribution of manuals, follow-up instructions) 3. Follow-up	1. Assess patient and caregiver self-management ability 2. Establish a wechat communication group 3. Work out a discharge plan 4. After discharge, the nursing staff formulated personalized rehabilitation training programs and guidance 5. Diversified health education 6. Dynamic assessment of patient training 7. Safe care at home 8. Follow-up tracking	Femoral shaft fracture	Before intervention, 2 weeks after intervention	⑨①①
Lu et al. (2023)	2023	73.92 ± 6.12	75.20 ± 6.97	53	50	1. Perioperative period (medication guidance, demonstration of functional exercises, dietary guidance) 2. Discharge (explanation of precautions) 3. Follow-up	1. Set up a multidisciplinary research group 2. Formulation of discharge plan 3. Identify and assess patients, establish archive 4. Strengthen osteoporosis health and safety management education 5. Intervention of fracture risk factors and guidance of postoperative functional exercise 6. Predischarge health education and safety guidance, Be ready for discharge 7. Follow-up tracking	Internal fixation of femoral neck in the elderly	After intervention, 1 month, 3 months, 6 months, 9 months after intervention	⑦⑨⑩
Ling, Sun & Qiu (2022)	2022	72.64 ± 9.13	73.09 ±9.20	43	43	1. During hospitalization (disease knowledge, postoperative precautions, exercise and lifestyle guidance, observation of fracture site) 2. At discharge (distribution of manuals, follow-up notification) 3. Follow-up	1. Set up a multidisciplinary research group 2. Develop discharge plans 3. Hospital intervention (Assessment of self-management ability of patients and caregivers, Establish a wechat communication group) 4. Personalized health education and dynamic evaluation 5. Personalized care after discharge (The discharge needs of patients and their families were assessed again before discharge) 6. Patients’ mastery was followed up, Give dynamic guidance	Femur fracture in the elderly	Before, after and 3 months after intervention	②①①
Wang et al. (2023) ]	2023	65.25 ± 7.01		95	105	1. Perioperative period (psychological care) 2. Guidance (medication, education, follow-up)	1. Set up a multidisciplinary research group 2. Construction of discharge planning service scheme based on mind map 3. Homogenized training and assessment by head nurses 4. Systematic evaluation of patients 5. Diversified health education (Instruct patients and caregivers to carry out rehabilitation exercise) 6. Self-care and safety management assessment and guidance 7. Identify referral sites to assist with referrals 8. Follow-up tracking	Fragility fracture of hip in the elderly	Before intervention, after intervention, 1 month and 3 months after intervention	①②③④⑥
Peng, Xu & Chen (2023)	2023	58.58 ± 9.34	59.76 ± 9.08	41	42	1.Admission (education, preoperative preparation) 2. Intraoperative (environmental adjustment, temperature maintenance) 3. Postoperative (monitoring, diet, pain management, psychological care) 4. Discharge (follow-up emphasis) 5. Follow-up	1. The head nurse formed a multidisciplinary research team 2. Construct discharge planning service scheme based on motivational behavior change theory 3. Motivational intervention (Evaluate patients’ basic information and rehabilitation motivation, and encourage rehabilitation exercise) 4. Behavioral intervention (Pain, nutrition, comfort, functional exercise and complication management) 5. Individualized health education for patients and their caregivers 6. Assess discharge readiness and assist with discharge 7. Follow-up tracking	Femur fracture	Before intervention, after intervention, 12 weeks after intervention	①④
Wang (2021)	2021	–	–	43	40	1. Admission (assessment, ward introduction) 2. Preoperative (blood preparation, skin preparation, skin test, dietary education) 3. Postoperative (monitoring, guidance on diet/turning/exercise, complication prevention) 4. Discharge (medication, rehabilitation guidance, follow-up)	1. Set up a multidisciplinary research group, Identify a navigation nurse 2. To develop a guide for postoperative health education 3. Formulation of discharge plan service process 4. Assessment of discharge needs 5. Make discharge plan (Identify the patient’s problem and formulate with patient and primary caregiver) 6. Implementation plan (Pain, functional exercise, safety management (fall prevention), caregiver health guidance, identification of referral sites to assist referral, follow-up tracking) 7. Evaluation of effect	Fragility fracture of hip in the elderly	Before intervention, after intervention, 1 month and 3 months after intervention	①③④⑤⑥⑦⑧

**Notes.**

① Self-care ability, ② quality of life, ③ hip function, ④ readiness for hospital discharge, ⑤ average length of stay, ⑥ complication, ⑦ readmission rate, ⑧ satisfaction, ⑨ compliance, ⑩ incidence of falls, and ①① lower limb function.

### Risk of bias in the included studies

**Figure 2:**
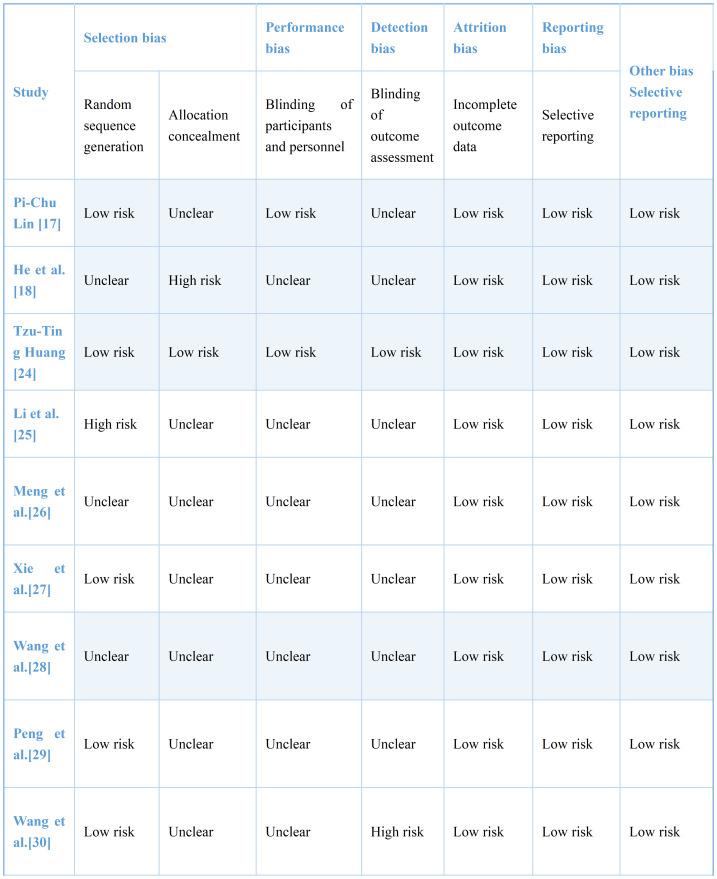
Risk of bias graph.

The methodological quality of the nine included RCTs was evaluated using the Cochrane Risk of Bias tool (Fig. 2). Among these, five studies (Lin et al., 2009; Huang & Liang, 2005; Ling, Sun & Qiu, 2022; Peng, Xu & Chen, 2023; Wang, 2021) provided explicit descriptions of their randomization methods. Huang & Liang (2005) provided detailed information on both allocation concealment and the blinding of outcome assessors. All the studies included studies provided complete outcome data, accounting for accounted-for follow-up losses. Based on these evaluations, the quality of one study (Huang & Liang, 2005) was rated as grade A, with the risk of bias for all seven items being low.

### Synthesis results

#### Readiness for hospital discharge

**Figure 3:**
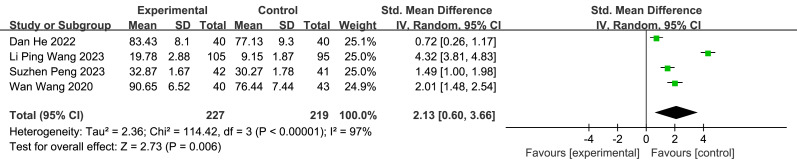
Influence of DPS on the discharge readiness of patients with hip fractures.

Four studies (Dan et al., 2022; Wang et al., 2023; Peng, Xu & Chen, 2023; Wang, 2021) involving 318 patients evaluated the impact of DPS on discharge readiness, using the Readiness for Hospital Discharge Scale (RHDS) as the measurement tool. The meta-analysis revealed a significant positive effect of DPS (SMD = 2.13, 95% CI [0.60–3.66], *P* = 0.006). However, considerable heterogeneity was observed (I^2^ = 97%, *P* < 0.001). A sensitivity analysis, excluding the study by Peng, Xu & Chen (2023), revealed a significant result (*P* = 0.006) and substantially reduced heterogeneity (I^2^ = 81%), suggesting that study might be a primary source of variability. This heterogeneity may be attributable to differences in intervention components, such as the involvement of a psychologist. The forest plot is presented in Fig. 3.

##### Subgroup analysis: “Non-osteoporotic fracture group” and “osteoporotic fracture group”.

**Figure 4:**
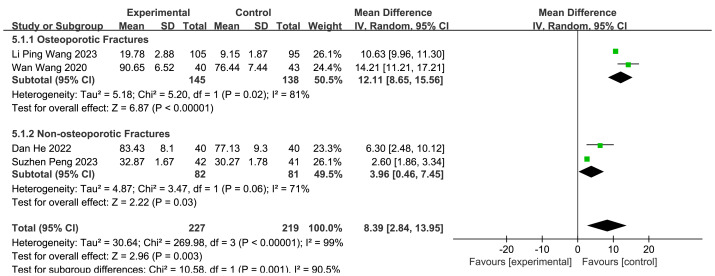
Subgroup analysis of the impact of different fracture types on discharge readiness in patients with hip fractures.

Owing to the numerous sources of heterogeneity, given the potential influence of fracture aetiology on recovery, a subgroup analysis was performed on the basis of the type of hip fracture (*e.g.*, osteoporotic or non-osteoporotic, such as traumatic) to better understand its impact (Shepperd et al., 2013). The analysis included the same four studies (Dan et al., 2022; Wang et al., 2023; Peng, Xu & Chen, 2023; Wang, 2021). The results revealed a statistically significant difference between subgroups (*P* = 0.001). The positive effect of DPS on discharge readiness was evident in both the non-osteoporotic fracture group (MD = 3.96, 95% CI [0.46–7.45], *P* = 0.03; I^2^ = 71%) and the osteoporotic fracture group (MD = 12.11, 95% CI [8.65–15.56], *P* = 0.02; I^2^ = 81%), with a more pronounced effect observed in the latter (Fig. 4).

#### Self-care ability (Barthel Index)

**Figure 5:**
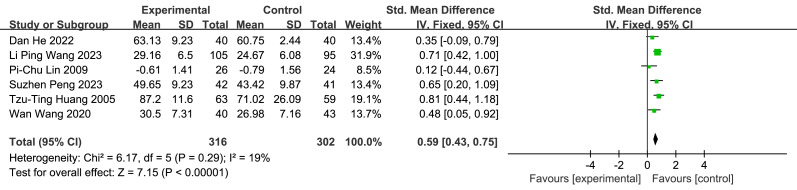
Effects of DPS on the self-care ability of patients with hip fractures.

Six studies (Lin et al., 2009; Dan et al., 2022; Huang & Liang, 2005; Wang et al., 2023; Peng, Xu & Chen, 2023; Wang, 2021) (*n* = 511) reported on self-care ability using the Barthel Index (BI). A pooled analysis conducted under a fixed-effects model (I^2^ = 19%, *P* = 0.290) indicated that DPS significantly improved patients’ self-care ability postoperatively, with a moderate effect size (SMD = 0.59, 95% CI [0.43–0.75], *P* < 0.001) (Fig. 5).

#### Hip function (Harris Hip Score)

**Figure 6:**
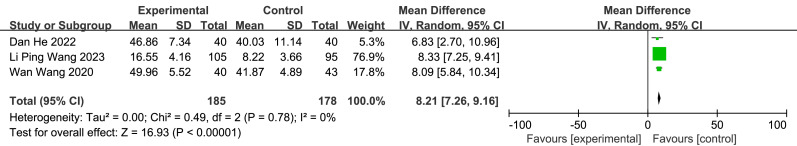
Impac t of DPS on hip function in patients with hip fracture.

Three studies (Dan et al., 2022; Wang et al., 2023; Wang, 2021) (*n* = 232) assessed hip functional recovery using the Harris Hip Score (HHS). No heterogeneity was detected (I^2^ = 0%, *P* = 0.780). The meta-analysis demonstrated that compared with the control group, patients who received DPS experienced significant improvement in hip function, with a mean improvement of 8.21 points (95% CI [7.26–9.16]; *P* < 0.001) compared to the control group (Fig. 6).

#### Lower limb motor function (Fugl–Meyer assessment)

**Figure 7:**
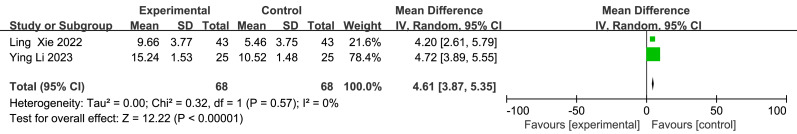
Impact of DPS on lower limb motor function in patients with hip fractures.

Two studies (Ying, Pan & Qiu, 2023; Ling, Sun & Qiu, 2022) (*n* = 142) evaluated lower limb motor function *via* the Fugl–Meyer Assessment Scale (FMAS). The studies were homogeneous (I^2^ = 0%, *P* = 0.57). The combined results revealed a significant benefit from DPS, with a mean difference of 4.61 points (95% CI [3.87–5.35]; *P* < 0.001) in favour of the intervention group (Fig. 7).

#### Lower limb balance function (Berg Balance Scale)

**Figure 8:**
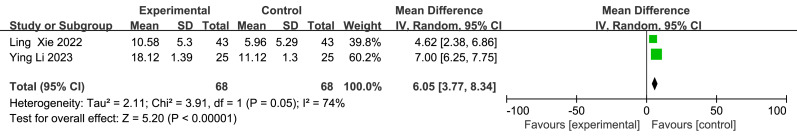
Effects of DPS on lower limb balance function in patients with hip fracture.

The same two studies (Ying, Pan & Qiu, 2023; Ling, Sun & Qiu, 2022) (*n* = 142) measured balance using the Berg Balance Scale (BBS). A random-effects model was utilized because of significant heterogeneity (I^2^ = 74%, *P* = 0.05), possibly due to differences in patient baselines or postdischarge support. Despite this variability, DPS was associated with a significant improvement in balance function (MD = 6.05, 95% CI [3.77–8.34]; *P* < 0.001) (Fig. 8).

### Complication rate

**Figure 9:**
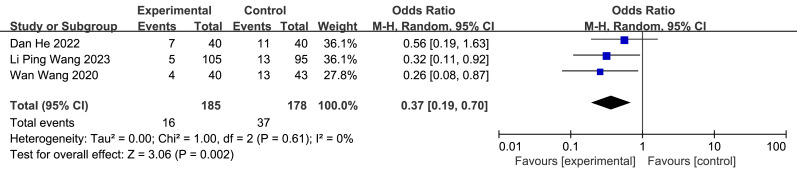
Impact of DPS on complications in patients with hip fractures.

Three studies (Dan et al., 2022; Wang et al., 2023; Wang, 2021) (*n* = 232) examined the incidence of complications such as infection and thrombosis. The results of the fixed-effects model analysis (I^2^ = 0%, *P* = 0.61) indicated that compared with the control group, the DPS group had a significantly lower risk of complications compared to the control group (OR = 0.37, 95% CI [0.19–0.70]; *P* = 0.002) (Fig. 9).

## Discussion

With respect to its impact on readiness for hospital discharge (RHD), the potential benefit of DPS remains suggestive but less definitive, primarily because of considerable variability across studies. This heterogeneity may be partially explained by patient characteristics and intervention components. For instance, the more pronounced effect observed in patients with osteoporotic fractures underscores the value of integrating targeted bone health management—such as individualized risk assessment and tailored treatment plans—into discharge planning, thereby addressing both immediate transition needs and long-term secondary prevention. Furthermore, the persistence of high heterogeneity (I^2^ = 81%) even after sensitivity analyses implies the influence of other unmeasured moderators. The notable reduction in heterogeneity upon excluding the study by Peng, Xu & Chen (2023), which featured a distinct psychological intervention, strongly indicates that variations in psychological support components such as strategies to alleviate anxiety and build coping skills initiated early during hospitalization may be a critical, yet underexplored, source of differential effectiveness. Therefore, while DPS demonstrates promise in enhancing RHD, future studies should prioritize the development of standardized protocols that explicitly incorporate and evaluate psychosocial and bone health elements, in addition to recruiting larger sample sizes, to clarify its true efficacy.

The positive impact of DPS on self-care ability underscores its role in bridging the gap between institutional care and independent living. This effect, achieved through structured education and sustained support (Van Dartel et al., 2021), appears to be most pronounced among patients with specific vulnerabilities. The null result in the large trial by Beaupre et al. (2005), in contrast to its significant benefit for high-risk individuals lacking social support, provides crucial insight: the efficacy of DPS may be optimized not through uniform application but through its capacity to identify and address specific psychosocial deficits. This perspective is strongly supported by existing evidence linking robust social support encompassing material, emotional, and informational resources to improved recovery outcomes (Zhang et al., 2021). The integration of targeted psychosocial assessments and interventions, potentially involving hospital social workers whose involvement is associated with reduced readmissions (Dimla et al., 2023), should therefore be a central component of future DPS frameworks. Such an approach ensures that care plans are tailored to individual needs and connected to community resources, thereby strengthening the external support systems vital for long-term recovery.

The results of this study demonstrate that DPS facilitates not only statistically significant but also clinically meaningful recovery of hip function, lower limb motor function, and balance. Specifically, the observed improvement in balance (BBS) of 6.05 points far exceeds the established minimal clinically important difference, indicating a substantial functional gain for patients (Li, Deng & Liu, 2021). Similarly, the effect on hip function (HHS SMD = 0.59) represents a medium effect size, confirming a meaningful clinical impact (Yusoff, Mulud & Mohammadnezhad, 2022). These findings align with those of Zheng & Tong (2019) and Khayya (2016) and are likely mediated through several mechanisms. Unlike conventional care, which typically provides discharge instructions within a limited time frame, DPS offers continuous, structured health education that is easier for patients and caregivers to understand and implement (Wang, 2021). This model facilitates personalized exercise guidance, active engagement, and consistent postdischarge follow-up for feedback and encouragement. It maximizes patient adherence to rehabilitation protocols (Ying, Pan & Qiu, 2023). The consistent positive findings across multiple functional domains emphasize the importance of a coordinated, continuous care pathway. To enhance functional outcomes, future clinical practice should prioritize ongoing health education and foster seamless collaboration between hospital and community teams to effectively support patients upon their return home

Postoperative complications following hip fractures are a significant cause of morbidity and mortality (Hori et al., 2020). Our analysis, which combines data from three studies, indicates that DPS can significantly reduce the incidence of these complications. This benefit is likely derived from the proactive and preventive nature of DPS, which facilitates early identification of high-risk patients and enables the implementation of individualized, preemptive nursing interventions (Lu, Kang & Li, 2019; Olsson, Karlsson & Ekman, 2007). However, the results were not consistent across all the studies. According to a study by Dan et al. (2022), which involved 80 patients with hip fractures, the implementation of discharge planning services did not significantly reduce the incidence of complications such as lower extremity deep vein thrombosis (DVT), urinary tract infections (UTIs), wound infections,or pressure sores compared with those in the control group. These findings highlight that complication risk is multifactorial and influenced by age, comorbidities, surgical factors, and caregiver vigilance (Hori et al., 2020; Lu, Kang & Li, 2019; Olsson, Karlsson & Ekman, 2007). Therefore, the effectiveness of DPS in preventing complications may depend on its ability to integrate comprehensive risk stratification and tailor interventions accordingly. Future programs should focus on conducting thorough patient assessments and strengthening collaborations with community and referral services to ensure continuous vigilance and intervention postdischarge, thereby bridging the gap in care continuity that often leads to adverse events.

### Limitations and recommendations

While this review presents promising evidence for DPS, it is important to exercise caution because of several limitations. The overall quality of the evidence is influenced by practical challenges such as blinding in DPS trials and clinical heterogeneity inherent in patient populations and intervention designs across studies. The generalizability of our findings may be limited because of the relatively small sample size (*n* = 861) and the underrepresentation of certain patient subgroups. Although our updated search did not yield any new eligible studies, the ever-evolving nature of evidence requires us to view these conclusions as current rather than definitive. Future research should prioritize large-scale, multicentre randomized controlled trials using standardized, well-defined DPS protocols. These studies focus on examining the effectiveness of integrating psychological support, structured social risk assessment, and personalized long-term bone health management into the DPS model. This approach will help to fully understand its potential and translate this knowledge into practical, holistic clinical practice.

## Conclusions

In conclusion, this meta-analysis demonstrated that discharge planning services can significantly improve key aspects of hip fracture recovery, such as enhancing self-care capabilities, and physical function, and reducing complications. However, the impact on discharge readiness remains uncertain because of significant heterogeneity and methodological variability among existing studies. Moving forwards, our focus should be on advancing the field by developing and evaluating standardized, multifaceted protocols for drug and psychosocial recovery. These findings should be robust in a clinical setting and specifically address social support and mental health determinants. Ultimately, the successful implementation of DPS depends on its ability to be tailored into a targeted, evidence-based strategy that ensures a safe and supported transition from hospital to home for all patients with hip fractures.

## What does this paper contribute to the wider global clinical community?

With respect to global clinical community, this study highlights the crucial role of DPS in ensuring a safe care transition for patients with brittle hip fractures. It directly affects patients’ self-care ability, functional decline, and the occurrence of complications postdischarge. It is important for clinicians and healthcare administrators to recognize that the success of DPS is not solely dependent on logistical planning but also actively addresses psychosocial vulnerabilities through support and intervention. Future research should focus on developing standardized, scalable, and cost-effective DPS frameworks that can be adaptable to various healthcare settings globally.

## Supplemental Information

10.7717/peerj.21270/supp-1 Supplemental Information 1 PRISMA checklist

10.7717/peerj.21270/supp-2 Supplemental Information 2 Systematic search strategy for hip fracture and discharge planning in the Cochrane Library database

10.7717/peerj.21270/supp-3 Supplemental Information 3 Systematic search strategy for hip fracture and discharge planning in the EMBASE database

10.7717/peerj.21270/supp-4 Supplemental Information 4 Systematic search strategies for hip fracture and discharge planning across EBSCO, CNKI, Wanfang, and VIP databases

10.7717/peerj.21270/supp-5 Supplemental Information 5 Systematic search strategy for hip fracture and discharge planning on the Ovid platform

10.7717/peerj.21270/supp-6 Supplemental Information 6 Systematic search strategy for hip fracture and discharge planning in the PubMed database

10.7717/peerj.21270/supp-7 Supplemental Information 7 Systematic search strategy for hip fracture and discharge planning in the Web of Science Core Collection

## Additional Information and Declarations

### Data Availability

The following information was supplied regarding data availability: This is a Systematic review/Meta-analysis. The data is available at Mendeley: https://data.mendeley.com/datasets/7r98hx35hm/3
