# Supplementary figures and images for: Discharge planning services for safe transition after hip fracture: a systematic review and meta-analysis of discharge readiness, functional recovery, and complication reduction
Source: PeerJ. 2026 Jun 11;14:e21270. doi: 10.7717/peerj.21270 (PMC13264975; doi:10.7717/peerj.21270)

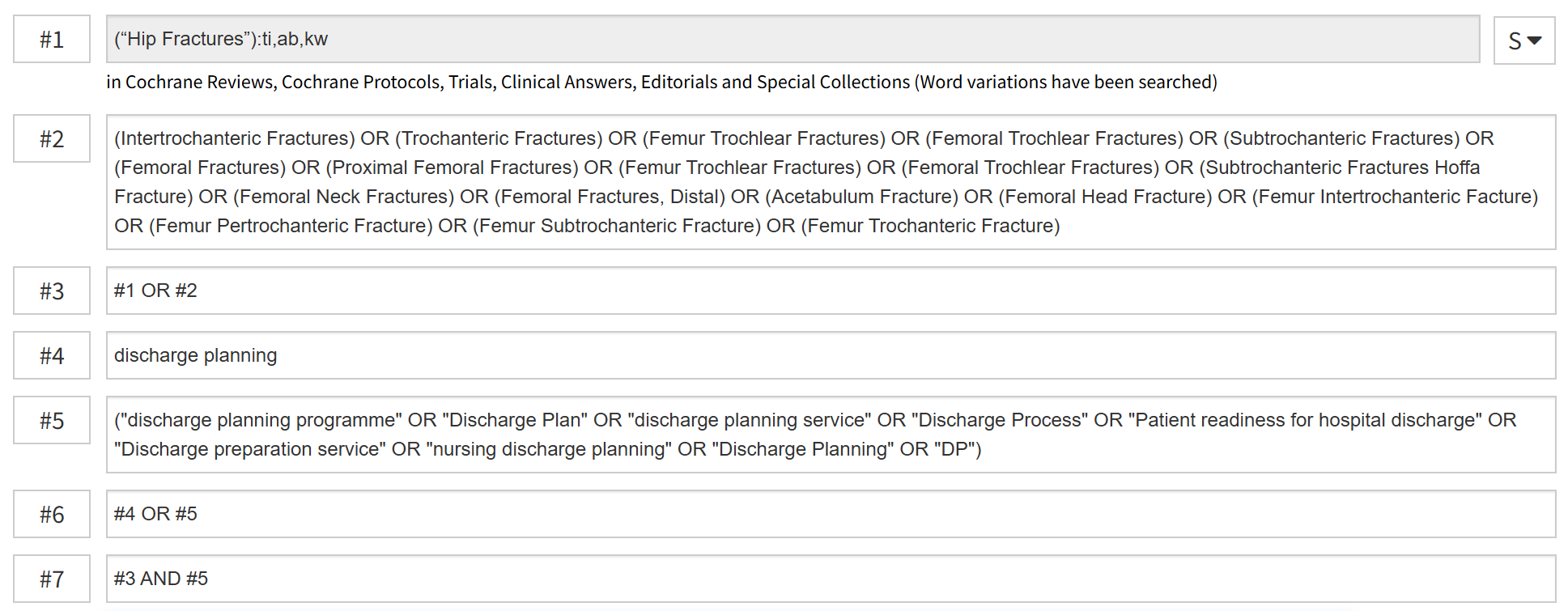

Supplement: Supplemental Information 2 [file peerj-14-21270-s002.png]

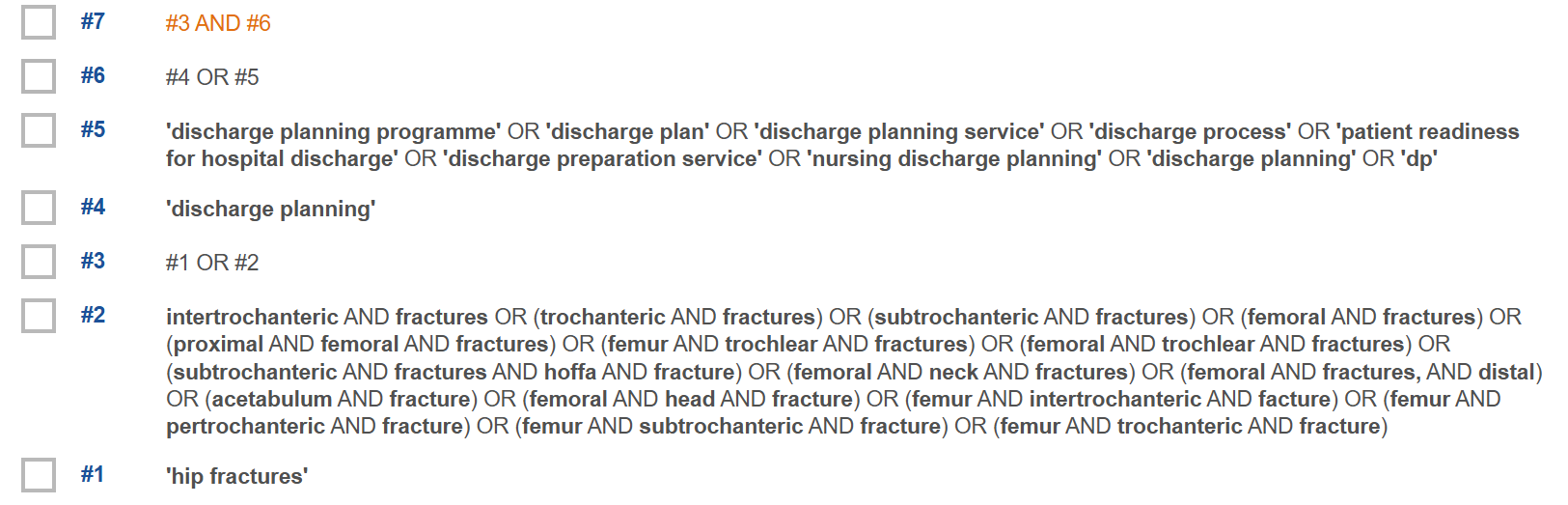

Supplement: Supplemental Information 3 [file peerj-14-21270-s003.png]

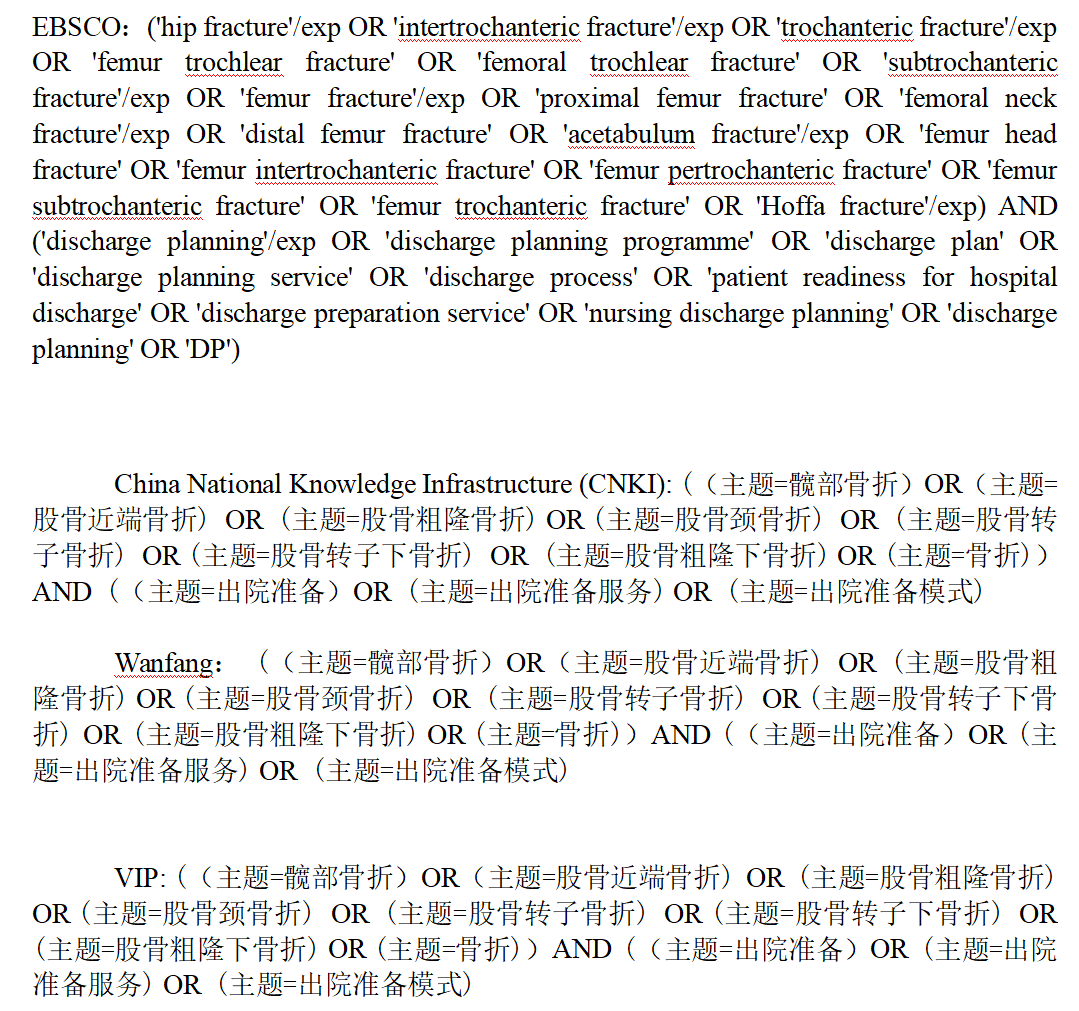

Supplement: Supplemental Information 4 [file peerj-14-21270-s004.png]

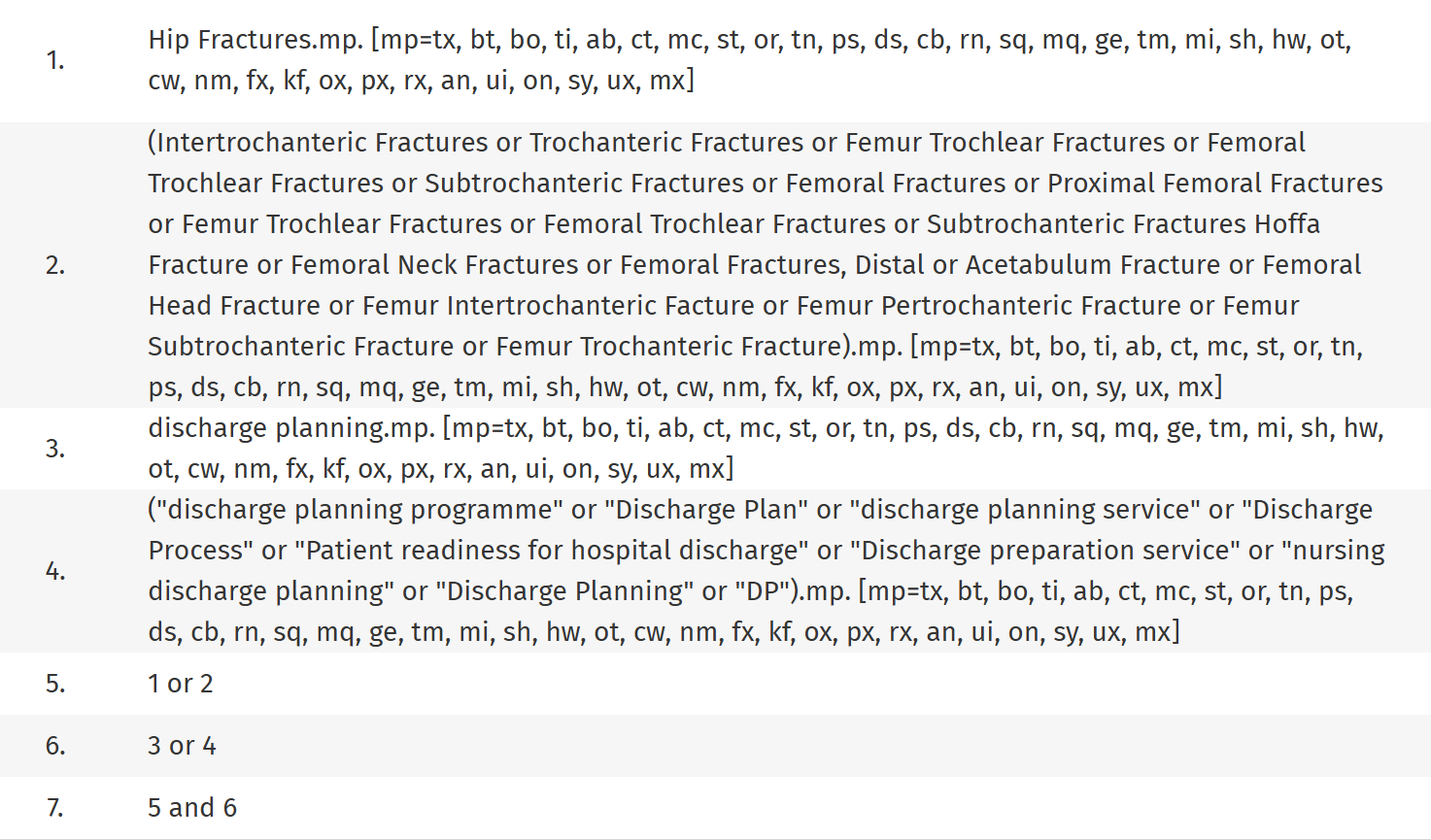

Supplement: Supplemental Information 5 [file peerj-14-21270-s005.png]

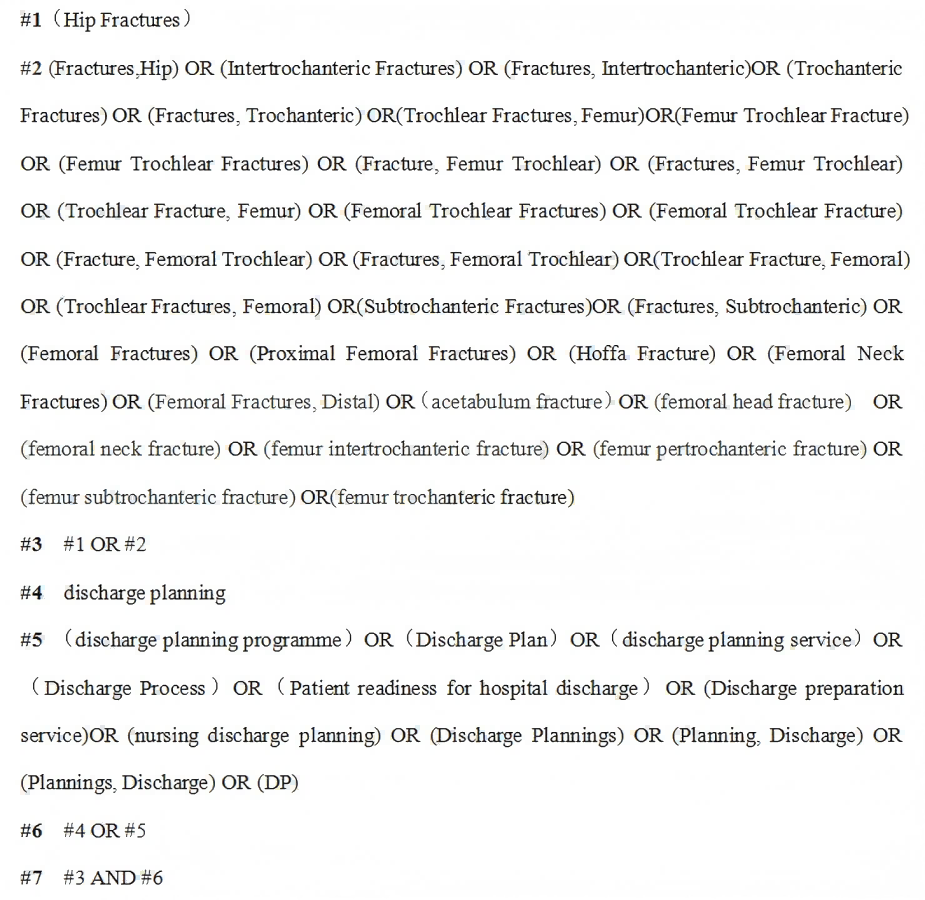

Supplement: Supplemental Information 6 [file peerj-14-21270-s006.png]

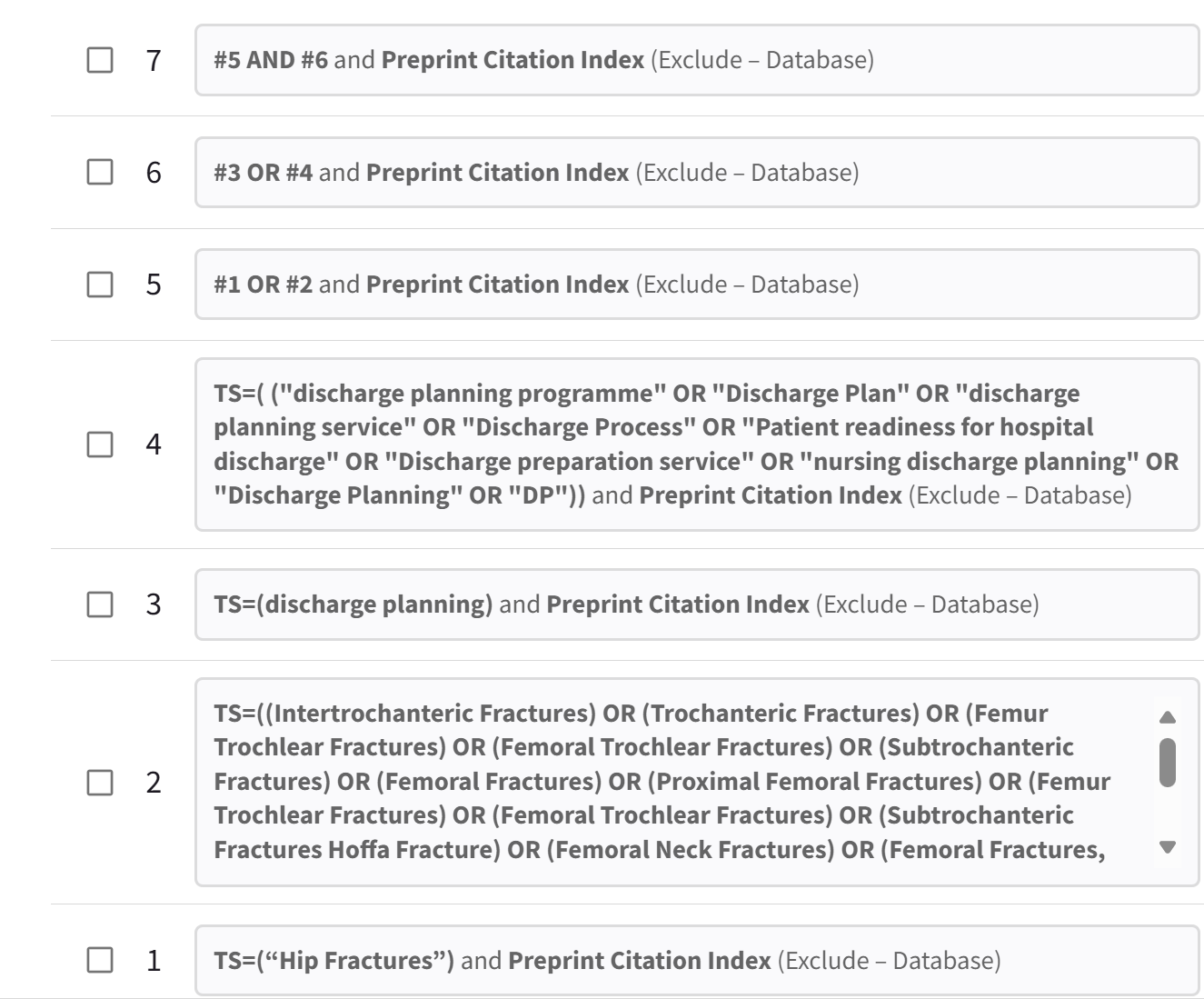

Supplement: Supplemental Information 7 [file peerj-14-21270-s007.png]
